# Supplementary material for: Genome-wide identification and expression profiling of odorant receptor genes in the malaria vector Anophelessinensis
Source: Parasit Vectors. 2022 Apr 23;15:143. doi: 10.1186/s13071-022-05259-x (PMC9034491; doi:10.1186/s13071-022-05259-x)
Supplement: Supplementary file 2 — Additional file 2: Table S2. Homology relationships of Anopheles sinensis with three mosquitoes and Drosophila melanogaster ORs. [file 13071_2022_5259_MOESM2_ESM.docx]

**Additional file2: Table S2.** Homology relationships of *Anopheles sinensis* with three mosquitoes and *Drosophila melanogaster* ORs.

| OR Name | *An. gambiae* homolog | AA identity | *Ae. aegypti* homolog | AA identity | *Cx. quinquefasciatus* homolog | AA identity | *D. melanogaster* homolog | AA identity |
| --- | --- | --- | --- | --- | --- | --- | --- | --- |
| AsOR1 | AgOR1 | 77%^a^ | - | - | CxOR1 | 42% | - | - |
| AsOR2 | AgOR2 | 88% | AaOR2 | 71% | CxOR121 | 69% | - | - |
| AsOR3 | AgOR3 | 86% | - | - | - | - | - | - |
| AsOR4 | AgOR4 | 45% | - | - | - | - | - | - |
| AsOR5 | AgOR5 | 83% | - | - | - | - | - | - |
| AsOR6 | AgOR6 | 59% | AaOR6 | 49% | CPIJ007691 | 54% | - | - |
| AsOrco | AgOR7 | 98% | AaOR7 | 89% | CxOR7 | 89% | DmOR83b | 77% |
| AsOR8 | AgOR8 | 85% | AaOR8 | 73% | CxOR118b | 72% | - | - |
| AsOR9 | AgOR9 | 56% | - | - | - | - | - | - |
| AsOR10 | AgOR10 | 90% | AaOR10 | 73% | CxOR2 | 73% | DmOR30a | 36% |
| AsOR11 | AgOR11 | 70% | AaOR11 | 56% | CxOR56a | 53% | - | - |
| AsOR13 | AgOR13 | 48% | AaOR14 | 32% | - | - | - | - |
| AsOR14 | AgOR14 | 44% | AaOR14 | 31% | - | - | - | - |
| AsOR16 | AgOR16 | 70% | AaOR15 | 31% | - | - | - | - |
| AsOR18^b^ | NA | NA | NA | NA | NA | NA | NA | NA |
| AsOR22 | AgOR22 | 67% | AAEL021097 | 54% | CxOR137 | 50% | - | - |
| AsOR23 | AgOR23 | 76% | AaOR23 | 46% | CxOR108 | 37% | - | - |
| AsOR24 | AGAP010507 | 64% | AAEL025135 | 35% | CxOR83 | 36% | - | - |
| AsOR28 | AgOR28 | 64% | - | - | - | - | - | - |
| AsOR29 | AgOR29 | 55% | - | - | CPIJ000543 | 30% | - | - |
| AsOR30 | AgOR30 | 52% | - | - | - | - | - | - |
| AsOR31 | AgOR31 | 75% | AaOR31 | 48% | CxOR82a | 47% | - | - |
| AsOR32 | AgOR32 | 52% | AaOR31 | 31% | - | - | - | - |
| AsOR33 | AgOR33 | 84% | AaOR33 | 35% | CxOR47b | 34% | - | - |
| AsOR34 | AgOR34 | 78% | AAEL003369 | 37% | CxOR94a | 36% | - | - |
| AsOR35 | AgOR35 | 72% | AaOR31 | 30% | CxOR82a | 30% | - | - |
| AsOR36a | AgOR36 | 65% | AaOR52 | 30% | - | - | - | - |
| AsOR36b | AgOR36 | 60% | - | - | - | - | - | - |
| AsOR37 | AgOR37 | 64% | AAEL003369 | 34% | CxOR71a | 36% | - | - |
| AsOR38 | AgOR80 | 71% | AaOR132.1 | 50% | CPIJ005662 | 49% | - | - |
| AsOR39 | AgOR39 | 67% | AaOR38 | 33% | CxOR10a | 33% | - | - |
| AsOR40 | AgOR40 | 72% | AaOR40 | 51% | CxOR91.2 | 47% | - | - |
| AsOR41 | AgOR41 | 57% | AaOR41 | 40% | CxOR63 | 39% | - | - |
| AsOR42 | AgOR42 | 56% | AaOR42 | 38% | CxOR161 | 37% | - | - |
| AsOR43 | AgOR43 | 59% | AaOR44 | 39% | CxOR83c | 38% | - | - |
| AsOR44 | AgOR44 | 60% | AaOR44 | 40% | CxOR83c | 39% | - | - |
| AsOR45 | AgOR45 | 41% | - | - | - | - | - | - |
| AsOR46 | AgOR46 | 54% | - | - | - | - | - | - |
| AsOR48 | AgOR48 | 67% | AAEL001303 | 33% | CxOR13a | 32% | - | - |
| AsOR49 | AgOR49 | 68% | AAEL001303 | 36% | CxOR13a | 37% | - | - |
| AsOR51 | AgOR51 | 49% | AaOR15 | 31% | CxOR4 | 30% | - | - |
| AsOR52 | AgOR52 | 47% | - | - | - | - | - | - |
| AsOR54 | AgOR54 | 54% | - | - | - | - | - | - |
| AsOR56 | AgOR56 | 53% | AaOR55 | 31% | CPIJ008023 | 30% | - | - |
| AsOR57 | AgOR57 | 52% | AaOR26 | 30% | CxOR9a | 30% | - | - |
| AsOR58 | AgOR58 | 68% | AaOR74 | 30% | CxOR110 | 39% | - | - |
| AsOR59 | AgOR59 | 53% | - | - | - | - | - | - |
| AsOR60 | AgOR60 | 40% | - | - | - | - | - | - |
| AsOR61a | AgOR61 | 58% | - | - | - | - | - | - |
| AsOR61b | AgOR61 | 61% | - | - | - | - | - | - |
| AsOR62 | AgOR62 | 67% | - | - | - | - | - | - |
| AsOR63 | AgOR63 | 60% | - | - | - | - | - | - |
| AsOR64^c^ | AgOR64 | 42% | - | - | - | - | - | - |
| AsOR66 | AgOR66 | 56% | AaOR66 | 49% | CxOR43a | 51% | - | - |
| AsOR68 | AgOR68 | 42% | AaOR70 | 31% | - | - | - | - |
| AsOR69 | AgOR69 | 55% | AaOR69 | 41% | CxOR60 | 35% | - | - |
| AsOR70 | AgOR70 | 55% | AaOR69 | 43% | CxOR60 | 39% | - | - |
| AsOR76 | AgOR76 | 54% | - | - | - | - | - | - |
| AsOR77 | AgOR77 | 62% | - | - | - | - | - | - |

a: Only those with amino acid consistency higher than 30% were provided; b: It is speculated that *AsOR18* is a pseudogene, so homology analysis was not done; c: *AsOR64* was provisionally annotated as an incomplete gene. NA: not analysis; - indicates no homology, or the amino acid identity is less than 30%.
