# Supplementary material for: Genome-wide identification and expression profiling of odorant receptor genes in the malaria vector Anophelessinensis
Source: Parasit Vectors. 2022 Apr 23;15:143. doi: 10.1186/s13071-022-05259-x (PMC9034491; doi:10.1186/s13071-022-05259-x)
Supplement: Supplementary file 4 — Additional file 4: Figure S1. The motifs of AsORs obtained from the MEME analysis. [file 13071_2022_5259_MOESM4_ESM.ppt]

## Slide 1
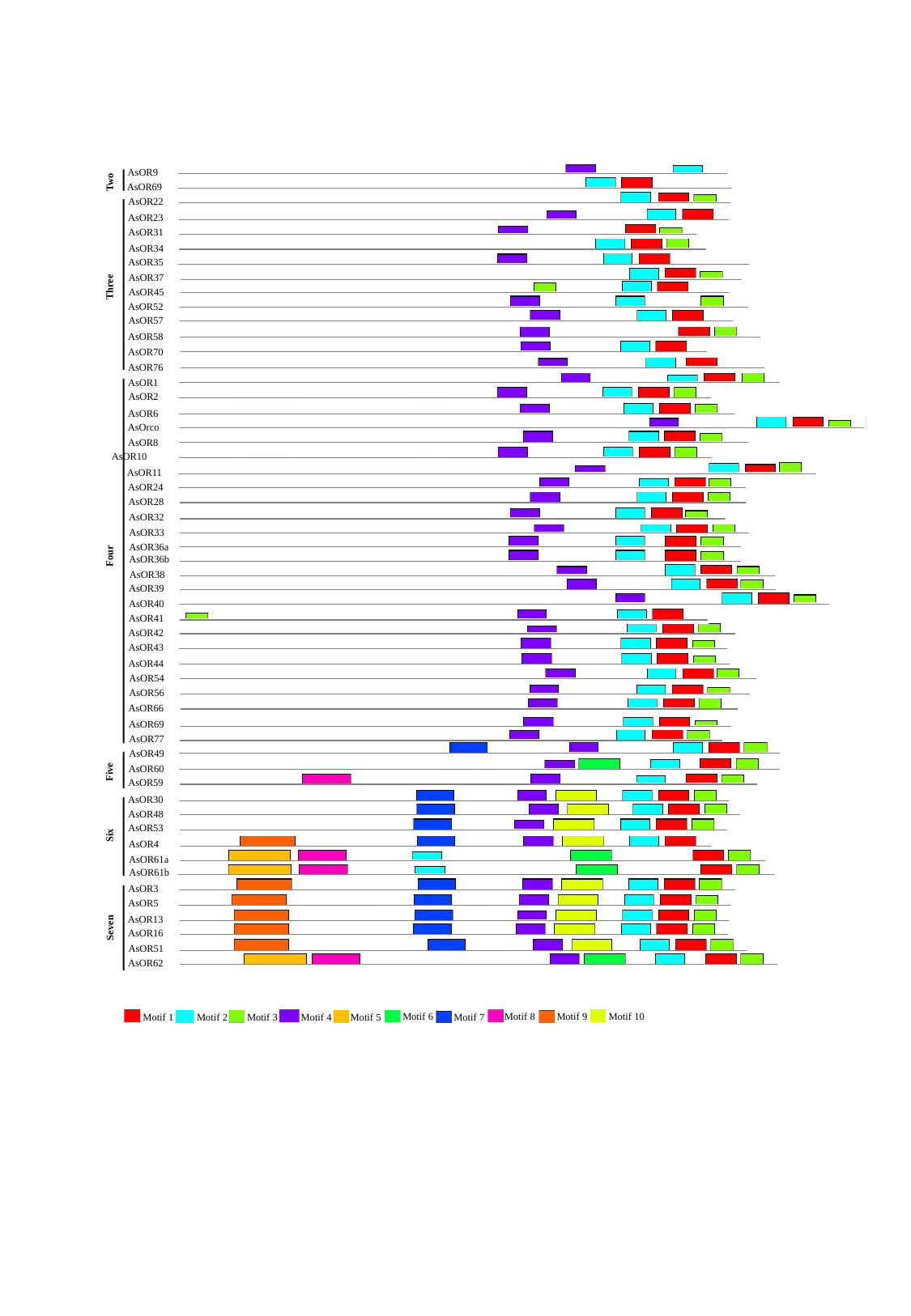

Two
AsOR9
AsOR69
AsOR22
AsOR23
AsOR31
AsOR34
AsOR35
Three
AsOR37
AsOR45
AsOR52
AsOR57
AsOR58
AsOR70
AsOR76
AsOR1
AsOR2
AsOR6
AsOrco
AsOR8
AsOR10
AsOR11
AsOR24
AsOR28
AsOR32
Four
AsOR33
AsOR36a
AsOR36b
AsOR38
AsOR39
AsOR40
AsOR41
AsOR42
AsOR43
AsOR44
AsOR54
AsOR56
AsOR66
AsOR69
AsOR77
Five
AsOR49
AsOR60
AsOR59
AsOR30
AsOR48
Six
AsOR53
AsOR4
AsOR61a
AsOR61b
AsOR3
Seven
AsOR5
AsOR13
AsOR16
AsOR51
AsOR62
Motif 8
Motif 10
Motif 9
Motif 6
Motif 1
Motif 7
Motif 2
Motif 5
Motif 3
Motif 4
