# Supplementary figures and images for: Genome-wide identification and expression profiling of odorant receptor genes in the malaria vector Anophelessinensis
Source: Parasit Vectors. 2022 Apr 23;15:143. doi: 10.1186/s13071-022-05259-x (PMC9034491; doi:10.1186/s13071-022-05259-x)

## Slide 1
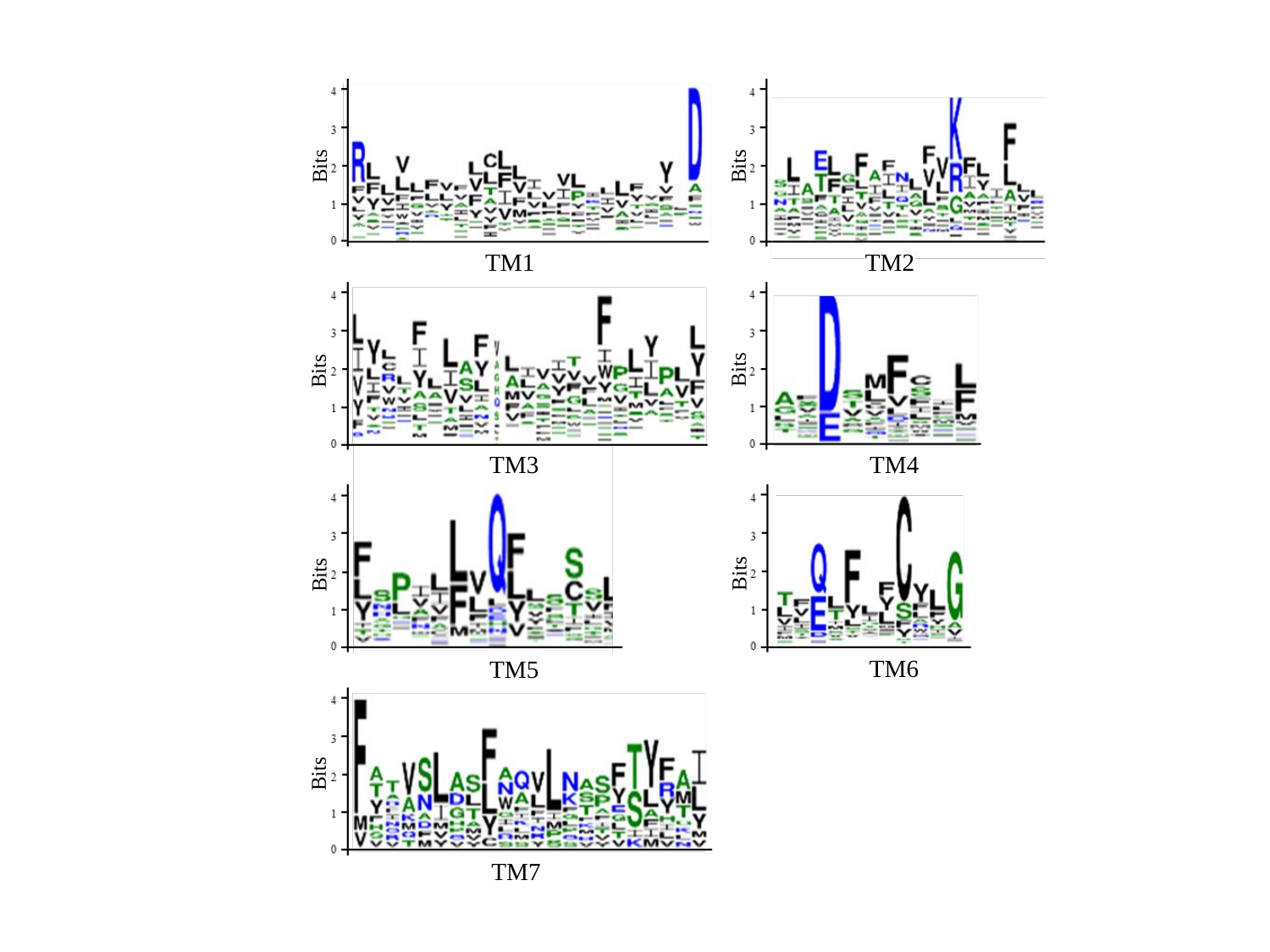

TM2
TM1
TM4
TM3
TM6
TM5
TM7
Bits
Bits
Bits
Bits
Bits
Bits
Bits

Supplement: Supplementary file 5 — Additional file 5: Figure S2. Sequence logo plot of transmembrane region of AsORs. [file 13071_2022_5259_MOESM5_ESM.ppt]

## Slide 1
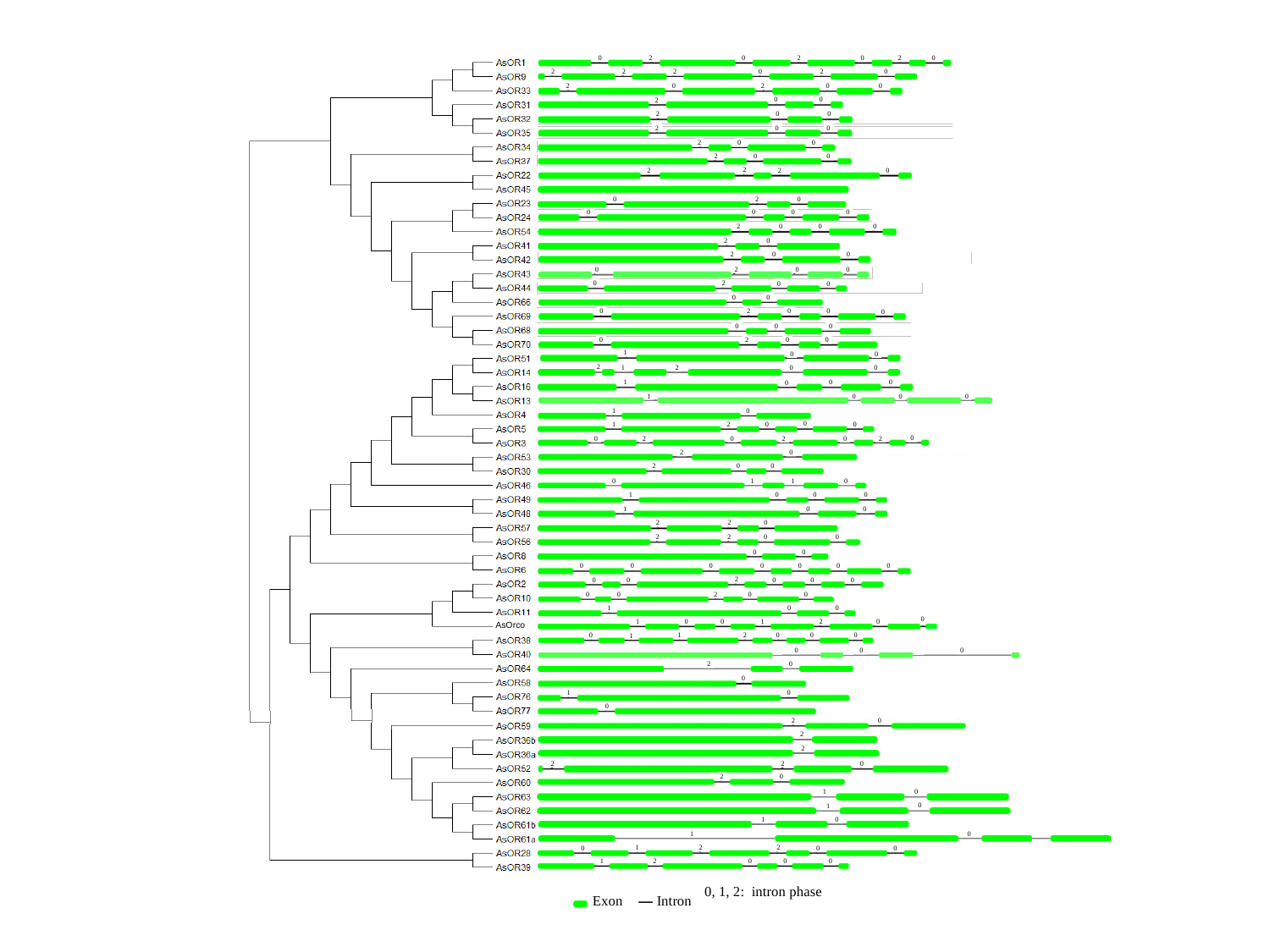

0, 1, 2: intron phase
Intron
Exon
2
0
0
2
2
0
0
2
2
0
0
2
2
0
2
2
0
0
0
0
2
0
0
2
2
0
0
2
0
0
2
0
0
2
2
0
2
0
0
2
0
0
0
0
0
0
0
2
0
2
0
0
2
2
0
0
0
0
2
0
0
0
0
0
2
0
0
0
0
0
0
0
2
0
0
1
0
0
2
0
0
2
1
0
1
0
0
0
0
1
0
0
1
0
0
0
1
2
0
0
2
0
0
2
2
2
0
0
2
0
1
1
0
0
0
0
1
0
1
0
0
2
0
2
2
2
0
0
0
0
0
0
0
0
0
0
0
2
0
0
0
0
0
2
0
0
0
0
1
0
0
0
0
0
2
1
0
1
1
0
0
2
0
0
1
0
0
0
0
2
0
1
0
0
2
0
2
2
0
2
2
2
0
0
1
0
1
1
0
1
0
1
2
2
0
0
0
1
0
0
0
2
AsOrco

Supplement: Supplementary file 6 — Additional file 6: Figure S3. Phylogenetic and gene structure analysis of AsOR genes. The unrooted maximum-likelihood phylogenetic tree was constructed on conserved domain under the model of JTT + I + G with 1000 bootstrap replicates. Gene structures were analyzed using the online server Gene Structure Display Server. Boxes in green were exons. Solid lines represent introns. Number 0, 1 and 2 represent intron phases. [file 13071_2022_5259_MOESM6_ESM.ppt]

## Slide 1
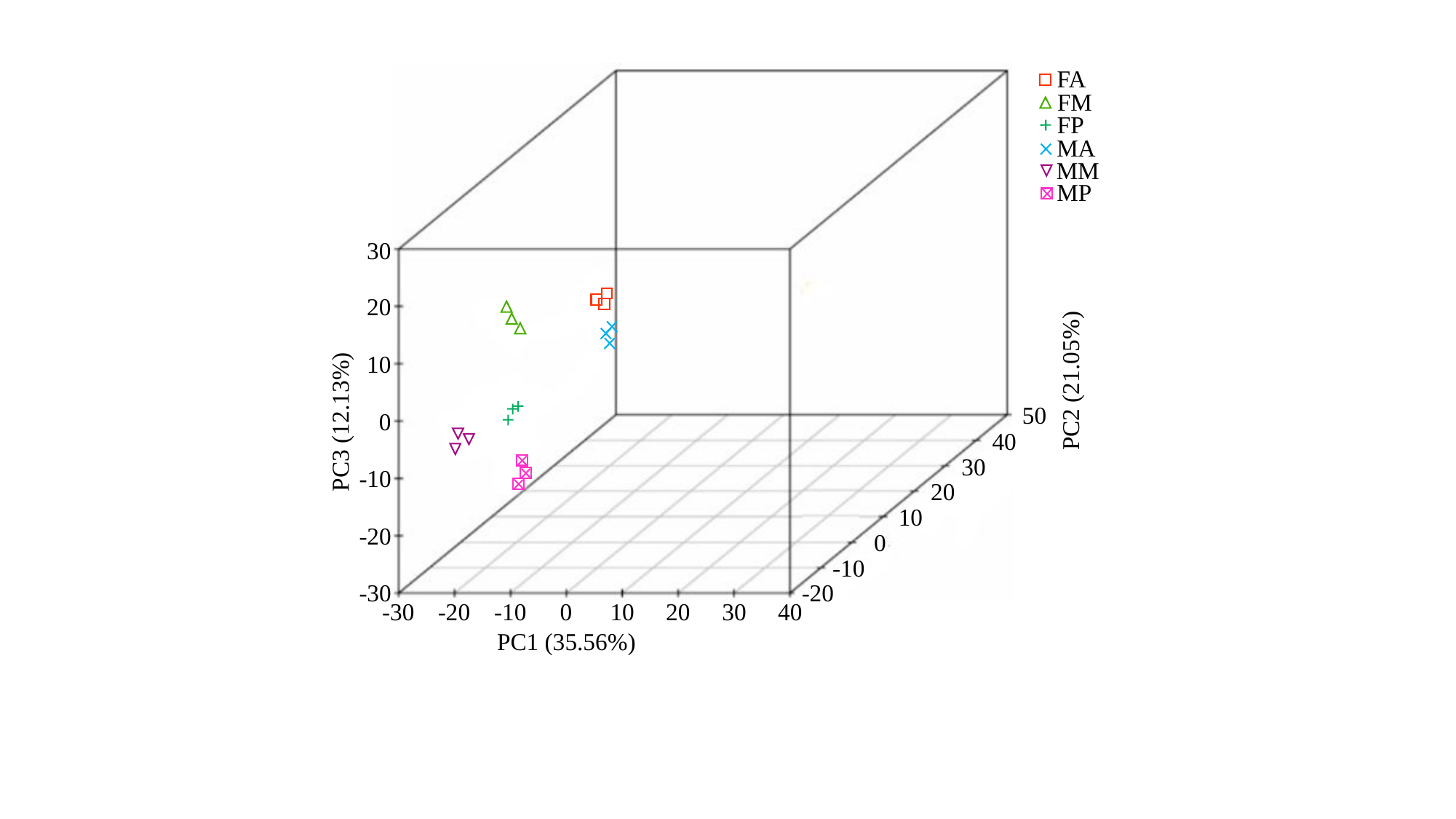

FA
FM
FP
MA
MM
MP
30
20
10
PC2 (21.05%)
50
0
PC3 (12.13%)
40
30
-10
20
10
-20
0
-10
-30
-20
-30
-20
-10
0
10
20
30
40
PC1 (35.56%)

Supplement: Supplementary file 8 — Additional file 8: Figure S4. S4 RNA-seq correlation check. FA: female antennae; FP: female proboscis; FM: female maxillary palps; MA: male antennae; MP: male proboscis; MM: male maxillary palps. [file 13071_2022_5259_MOESM8_ESM.ppt]

## Slide 1
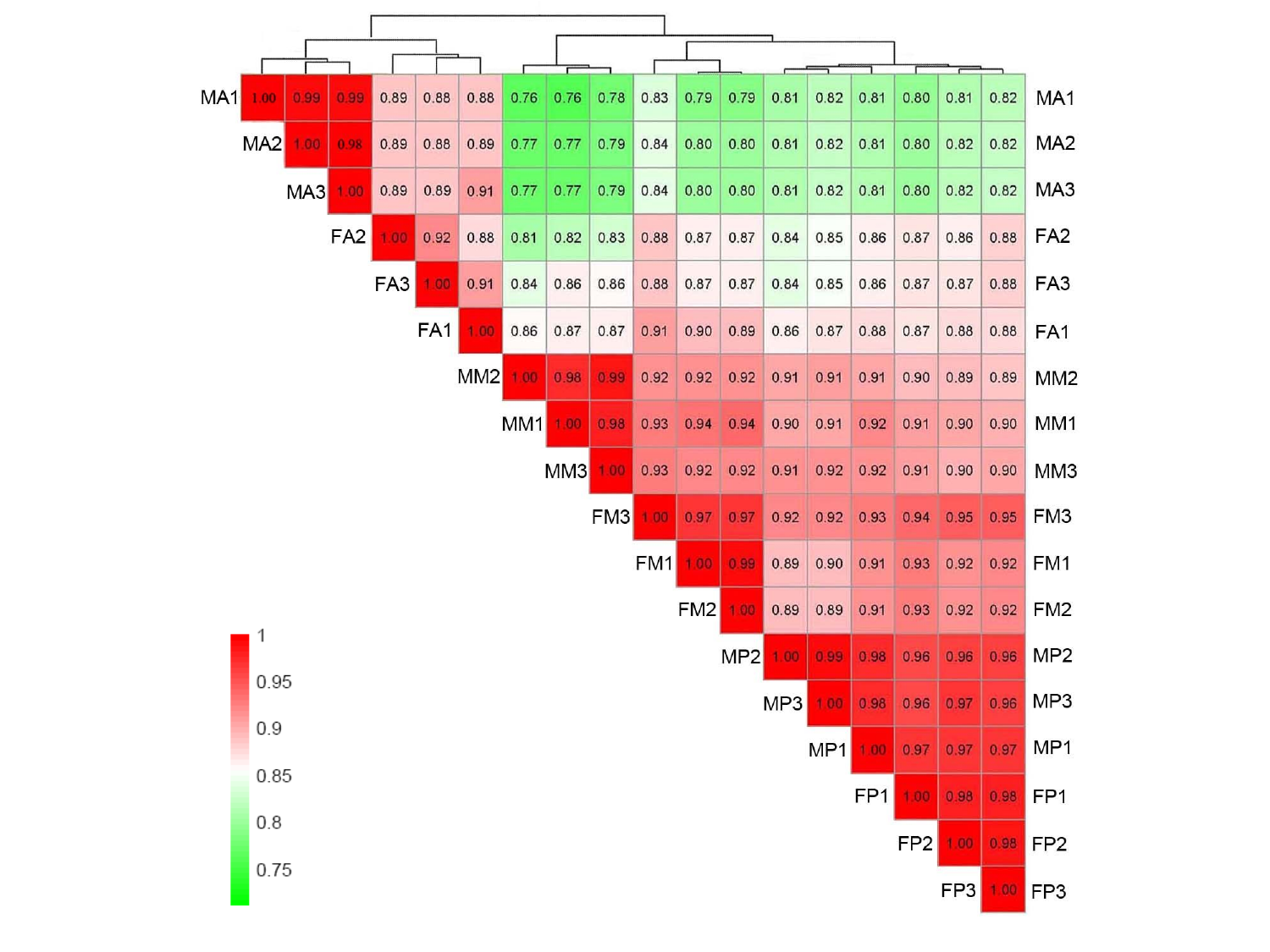

Supplement: Supplementary file 9 — Additional file 9: Figure S5. The correlation of 6 transcriptomes was analyzed using Euclidean Distance. FA: female antennae; FP: female proboscis; FM: female maxillary palps; MA: male antennae; MP: male proboscis; MM: male maxillary palps. [file 13071_2022_5259_MOESM9_ESM.ppt]

## Slide 1
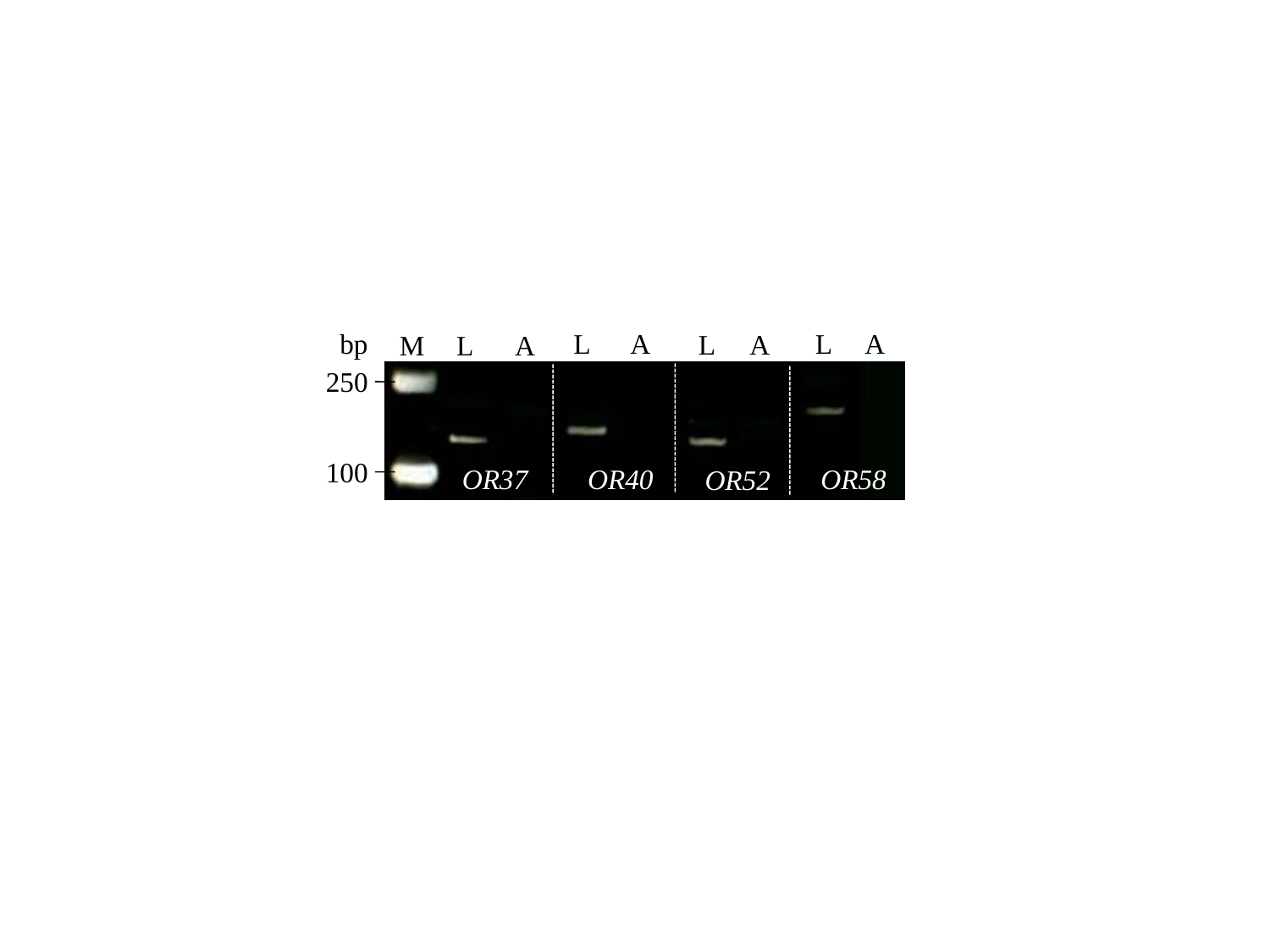

L
A
L
A
L
A
L
A
250
100
OR40
OR37
OR58
OR52
M
bp

Supplement: Supplementary file 13 — Additional file 13: Figure S6. The semi quantitative PCR results of OR genes. The total RNA was extracted from larvae or 3-day- old adults and was reverse transcribed into the first-strand cDNA using the SuperScript III RT Kit (Invitrogen, Carlsbad, CA, USA). These cDNAs were used as templates and semi quantitative PCR was performed. The primers were designed using Primer 5.0 and are listed in Additional file 1: Table S1. M: marker; L: larvae; A: adult. [file 13071_2022_5259_MOESM13_ESM.ppt]
