# Supplementary material for: Genome-wide identification and expression profiling of odorant receptor genes in the malaria vector Anophelessinensis
Source: Parasit Vectors. 2022 Apr 23;15:143. doi: 10.1186/s13071-022-05259-x (PMC9034491; doi:10.1186/s13071-022-05259-x)
Supplement: Supplementary file 11 — Additional file 11: Table S6. The chemosensory genes identified from the six transcriptomes. [file 13071_2022_5259_MOESM11_ESM.docx]

Additional file 11: Table S6. The chemosensory genes identified from the six transcriptomes

| Type | FA | FP | FM | MA | MP | MM |
| --- | --- | --- | --- | --- | --- | --- |
| CSPs | 4(3)* | 4(4) | 4(3) | 4(2) | 4(4) | 4(2) |
| OBPs | 17(14) | 19(12) | 18(12) | 18(14) | 16(11) | 12(5) |
| ORs | 32(25) | 24(7) | 23(8) | 33(20) | 17(8) | 15(3) |
| IRs | 31(16) | 35(11) | 33(4) | 33(21) | 32(11) | 31(4) |
| GRs | 34(15) | 34(19) | 31(11) | 31(17) | 33(21) | 26(10) |
| Total | 118(73) | 116(53) | 109(38) | 119(74) | 102(55) | 88(24) |

*The number outside the bracket indicates the number of chemosensory genes identified in the transcriptome, while the value inside the bracket indicates the number of genes expressed in transcriptome, with FPKM > 0.1 as the expression threshold.
