# Supplementary material for: Genome-wide identification and expression profiling of odorant receptor genes in the malaria vector Anophelessinensis
Source: Parasit Vectors. 2022 Apr 23;15:143. doi: 10.1186/s13071-022-05259-x (PMC9034491; doi:10.1186/s13071-022-05259-x)
Supplement: Supplementary file 12 — Additional file 12: Table S7. Statistical table of chemosensory genes in different expression levels. [file 13071_2022_5259_MOESM12_ESM.docx]

Additional file 12: Table S7. Statistical table of chemosensory genes in different expression levels

| FPKM Interval | 0~0.1 | 0.1~1 | 1~3 | 3~15 | 15~60 | >60 |
| --- | --- | --- | --- | --- | --- | --- |
| FA | 73(50.00%) | 43(29.45％) | 15(10.27％) | 8(5.48%) | 4(2.74%) | 3(2.05%) |
| FP | 92(63.01%) | 42(28.76％) | 10(6.85％) | 2(1.37％) | 0 | 0 |
| FM | 108(73.97%) | 27(18.49％) | 6(4.11%) | 5(3.42％) | 0 | 0 |
| MA | 72(49.32%) | 47(32.19％) | 14(9.59%) | 11(7.53％) | 1(0.68%) | 0 |
| MP | 91(62.33) | 39(26.71％) | 13(8.90％) | 3(2.05％) | 0 | 0 |
| MM | 121(82.88%) | 20(13.70％) | 3(2.05％) | 2(1.37%) | 0 | 0 |
